# Markers of Sleep Disordered Breathing and Diabetes Mellitus in a Multiethnic Sample of US Adults: Results from the National Health and Nutrition Examination Survey (2005–2008)

**DOI:** 10.1155/2012/879134

**Published:** 2012-02-01

**Authors:** Charumathi Sabanayagam, Srinivas Teppala, Anoop Shankar

**Affiliations:** ^1^Department of Community Medicine, West Virginia University School of Medicine, Robert C. Byrd Health Sciences Center, Medical Center Drive, P.O. Box 9190, Morgantown, WV 26505-9190, USA; ^2^Singapore Eye Research Institute, Singapore National Eye Center, 11 Third Hospital Avenue, No. 05-00, Singapore 168751

## Abstract

We examined gender and ethnic differences in the association between sleep disordered breathing (SDB) and diabetes among 6,522 participants aged ≥20 years from the National Health and Nutrition Examination Survey 2005–08. SDB severity was defined based on an additive summary score including sleep duration, snoring, snorting, and daytime sleepiness. We found that the summary SDB score was significantly associated with diabetes after adjusting for potential confounders in the whole population. Compared to those without any sleep disturbance, the multivariable odds ratio (OR) (95% confidence interval (CI)) of diabetes among those with ≥3 sleep disturbances was 2.04 (1.46–2.87). In sex-specific analyses, this association was significant only in women (OR (95% CI) = 3.68 (2.01–6.72)) but not in men (1.10 (0.59–2.04)), *P*-interaction = 0.01. However, there were no ethnic differences in this association, *P*-interaction = 0.7. In a nationally representative sample of US adults, SDB was independently associated with diabetes only in women, but not in men.

## 1. Introduction

Sleep disordered breathing (SDB) and sleep apnea are associated with cardiovascular disease [1] and mortality [2]. It has been estimated that approximately 12–18 million adults are affected by SDB in the USA [3]. Recent studies conducted in the USA [4–10] and Europe [11–14] have shown an association between a variety of markers of SDB including snoring [4, 11, 15], daytime sleepiness [8, 12, 13], or sleep duration [5–7, 10, 14] and diabetes. However, some of these studies used either a single marker of SDB or were conducted in specific populations including men [6], occupational cohorts [4], or older adults [9].

It is not clear if there are gender or ethnic differences in the association between SDB and diabetes in the USA. SDB is often underevaluated and underdiagnosed in women [16]. Previous studies, mostly conducted in the Europe [9, 12, 13, 17], have reported gender differences in the association between SDB and diabetes. Two of these studies have reported an association only in women [12, 17] while one study has reported an association in only men [13]. In the USA, Enright et al. reported that snoring was associated with diabetes in older women but not in older men in the Cardiovascular Health Study [9]. African Americans and Hispanic Americans were shown to be disproportionately affected by both sleep disturbances [18] and diabetes [19]. In studies involving predominantly white samples, including the Sleep Heart Health Study (93% whites) [20] and the Wisconsin Sleep Cohort Study (96% whites) [21], SDB was found to be associated with diabetes, whereas SDB was not associated with diabetes in a predominantly African American and Hispanic population in a clinical study in Chicago [22]. In this context, we examined the association between several subjective markers of SDB including snoring, snorting, daytime sleepiness, and sleep duration and diabetes in a nationally representative sample of US adults after accounting for depression and other potential confounders. We also examined the association between markers of SDB and diabetes stratified by gender and race-ethnicity.

## 2. Methods

The data for this study is derived from the National Health and Nutrition Examination Survey (NHANES) 2005-2006 and 2007-2008. Detailed description of NHANES study design and methods is available elsewhere [23, 24]. In brief, the NHANES survey included a stratified multistage probability sample representative of the civilian noninstitutionalized US population. Selection was based on counties, blocks, households, and individuals within households, and included oversampling of non-Hispanic blacks and Mexican Americans in order to provide stable estimates of these groups. We restricted our study sample to participants aged greater than 20 years (age range 20–85 years). Questions on sleep were first included in the NHANES in 2005-06. Of the 10,914 participants with information on sleep variables, after excluding those with pregnancy (*n* = 393), prevalent cardiovascular disease (*n* = 1, 275), those with missing information on fasting or nonfasting glucose or sleep variables (*n* = 2, 109), and those included in the multivariable model (*n* = 615), 6,522 were available for the final analysis.

### 2.1. Outcome of Interest: Diabetes Mellitus

Diabetes was defined as a serum glucose ≥126 mg/dL after fasting for a minimum of 8 hours, a plasma glucose ≥200 mg/dL for those who fasted <8 hours before their NHANES visit, or glycosylated hemoglobin (HbA1c) ≥6.5%, a self-reported physician-diagnosed diabetes or current use of oral hypoglycemic medication or insulin [25]. Because NHANES does not collect information (e.g., c-peptide levels) to identify the type of diabetes, we did not distinguish between type 1 and type 2 diabetes. However, we believe that the majority of diabetes subjects in our sample are due to type 2 diabetes mellitus. Fasting plasma glucose levels were measured using hexokinase enzymatic method on Roche/Hitachi Modular P Chemistry Analyzer at the Fairview Medical Center Laboratory at the University of Minnesota, Minneapolis MN. Random plasma glucose levels were measured at the Collaborative Laboratory Services in Ottumwa, IA using the Beckman Synchron LX20 in 2007 and the Beckman Coulter UniCel DxC800 in 2008. Glycosylated hemoglobin measurements were performed on the Tosoh 2.2 Analyzer (Tosoh Medics, Inc., 347 Oyster Pt. Boulevard, Suite 201, So., San Francisco, Ca) in NHANES 2005-06 and on the Automated HPLC System Glycohemoglobin Analyzer (Tosoh Medics, Inc., 347 Oyster Pt. Blvd., Suite 201, So. San Francisco, Ca 94080) in NHANES 2007-08 at the University of Minnesota, Minneapolis Minnesota.

### 2.2. Assessment of Exposure

A questionnaire on sleep habits based on validated questions from previous epidemiological studies was introduced in NHANES from 2005 till 2008 [26]. SDB was assessed from a set of questions on sleep habits including “How much sleep do you usually get at night on weekdays or workdays?”, “In the past 12 months, how often did you snore while you were sleeping?”, “In the past 12 months, how often did you snort, gasp or stop breathing while you were asleep?”, and “In the past month, how often did you feel excessively or overly sleepy during the day?” Based on the responses to the above questions, we created four sleep variables: sleep duration, snoring, snorting, and daytime sleepiness. Sleep duration coded in hours was categorized into ≤5, 6, 7, 8, and ≥9 h. Snoring and snorting variables were categorized into never or rare, occasional (3-4 nights/week), and frequent (5 or more nights/week). Daytime sleepiness was categorized into never or rare, sometimes (2–4 times/month), and often or almost always (5 or more times/month).

Based on the rationale that subjects with multiple SDB markers (e.g., snoring, snorting, daytime sleepiness) are likely to have more severe SDB, we developed an additive SDB summary score to examine the effect of cooccurrence of various SDB markers in relation to diabetes mellitus. For the summary score, we first dichotomized the individual variables based on their clinical significance and previous literature [10, 27, 28]. A score of 1 was assigned separately if the participants report sleep duration of ≤5, snoring at least 3-4 nights/week, snorting at least 3-4 nights/week and daytime sleepiness at least 5 times/month. The summary score ranged from 0 to 4 corresponding to no sleep disturbance to coexistence of all 4 sleep disturbances. We assessed diagnosed sleep apnea from the question, “Have you ever been told by a doctor or other health professional that you have a sleep disorder?” with dichotomous responses (“yes” or “no”).

### 2.3. Assessment of Covariates

Information on age, gender, race/ethnicity, smoking status, alcohol intake (g/day), level of education, history of diabetes and oral hypoglycemic intake or insulin administration, and antihypertensive medication use was obtained during a standardized questionnaire at home interview. Educational attainment was categorized into less than high school graduate, high school graduate, and more than high school graduate. Individuals who had smoked <100 cigarettes during their lifetime were considered never smokers, those who had smoked ≥100 cigarettes lifetime and currently not smoking were considered former smokers, and those who had smoked ≥100 cigarettes lifetime and currently smoking were considered current smokers. Current alcohol drinking was defined as consumption of ≥1 alcoholic drink in the past 12 months. Moderate physical activity was defined as engaging in moderate-intensity sports, fitness, or recreational activities that cause a small increase in breathing or heart rate such as brisk walking, bicycling, swimming, or golf for at least 10 minutes continuously in a typical week. Information on anthropometric, physical, and laboratory components was obtained during the medical examination center (MEC) examination. Body weight measured in pounds using a digital scale was converted to kilograms. Standing height was measured using a stadiometer and corrected to the nearest tenth of a centimeter [29]. Body mass index (BMI) was calculated as weight in kilograms divided by heights in meter squared. We defined overweight as BMI ≥ 25 kg/m^2^ and obesity as BMI ≥ 30 kg/m^2^ based on the WHO international classification [30] and the US Centers for Disease Control and Prevention guidelines [31]. Blood pressure (BP) was measured using a mercury sphygmomanometer, and an average of three measurements was taken as the systolic and diastolic BP value. Hypertension was defined as a systolic BP ≥ 140 mm Hg or a diastolic BP ≥ 90 mm Hg or a self-reported physician-diagnosed hypertension or current use of BP reducing medication [32]. Depression was assessed using the Patient Health Questionnaire (PHQ-9), a well-validated 9-item screening tool that asks questions about the frequency of symptoms of depression over the past 2 weeks [33]. Depression was defined as a PHQ-9 score of 10 or higher, a validated cut-point commonly used in clinical studies [33].

 Detailed description about the blood collection, processing, and quality control checks are provided in the Laboratory Procedures Manual [34, 35]. Serum C-reactive protein (CRP) was measured using latex-enhanced nephelometry. Total serum cholesterol was measured enzymatically using the Roche Hitachi 717 in 2005, Roche Hitachi 717 and 912 in 2006, and Roche Modular P chemistry analyzer in 2007-2008.

### 2.4. Statistical Analysis

We compared the characteristics of the study participants by diabetes status employing the chi square-test or analysis of variance as appropriate. We chose sleep duration of 7 hours as the reference category as previous studies have shown 7 hours of sleep to be associated with lower risk of CVD and mortality [36, 37]. We examined the association between categories of individual sleep variables, including snoring, snorting, daytime sleepiness, and sleep duration and diabetes in two multivariable models. In the first model, we adjusted for age (years) and sex (female, male). In the second model, we additionally adjusted for race-ethnicity (non-Hispanic whites, non-Hispanic blacks, Hispanic Americans, others), education (< high school, high school, > high school), smoking (never, former, current), current alcohol consumption (absent, present), physical activity (absent, present), body mass index (kg/m^2^), systolic blood pressure (mm Hg), depression (absent, present), CRP (mg/dL), and total cholesterol (mg/dL).

To examine the overall effect of SDB markers on diabetes, we created an additive summary SDB score and examined the association between this summary SDB score and diabetes in separate analyses. Tests for trend were performed using the categories of individual sleep variables and the summary score as an ordinal variable in the corresponding multivariable models. We tested for interactions between the summary SDB score and sex, race-ethnicity, and BMI categories by including cross-product interaction terms in the second multivariable model. To examine the consistency of the association we performed subgroup analyses stratified by sex, race-ethnicity, and BMI categories. All analyses were weighted to account for the unequal probabilities of selection, oversampling, and nonresponse using SUDAAN (version 8.0; Research Triangle Institute, Research Triangle Park, NC) and SAS (version 9.1.; SAS Institute, Cary, NC) software. In a supplementary analysis, we examined the association between diagnosed sleep apnea and diabetes.

## 3. Results

The prevalence of diabetes in the study population was 9%.  Table 1 shows the characteristics of the study population by diabetes status. Those with diabetes were more likely to be older, obese, had higher prevalence of hypertension, depression and higher levels of CRP, less likely to be non-Hispanic whites, above high school educated, current smokers, current drinkers, physically active, and had lower levels of total cholesterol.

**Figure 1:**
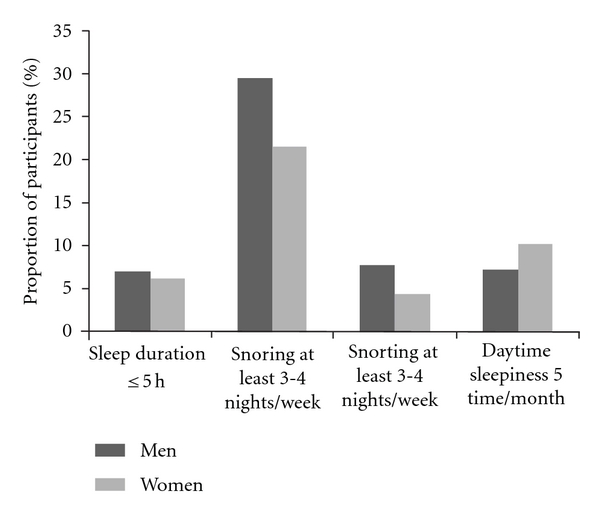
Proportion of participants by gender and by sleep disturbances.

Figure 1 shows the proportion of participants reporting sleep disturbances among men and women. Men were more likely to report snorting or snorting at least 3-4 nights/week (both *P* < 0.0001), and women were more likely to report daytime sleepiness at least 5 times/month (*P* = 0.0002).

Table 2 shows the association between the various sleep variables and diabetes mellitus. Compared to sleep duration of 7 h (referent), sleep duration ≤5 h was positively associated with diabetes in both the age-, sex-adjusted and the multivariable models. Occasional or frequent snoring (at least 3-4 nights/week), frequent snorting (at least 5 times/week), and frequent daytime sleepiness (at least 5 times/month) were significantly associated with diabetes in both the age-, sex-adjusted and the multivariable models. A significant graded association was observed between increasing number of sleep disturbances and diabetes (*P*-trend < 0.0001).

Table 3 shows the association between the summary SDB score and diabetes stratified by race-ethnicity. Consistent with the main results in Table 2, the association between summary SDB score and diabetes was found to be consistently present in all race-ethnic groups (*P*-interaction between SDB score and race-ethnicity = 0.7).  Table 4 shows the association between the summary SDB score and diabetes stratified by gender. The association between the summary SDB score and diabetes was significant in both men and women in the age-adjusted model, but in the multivariable model it was significant in women whereas it was substantially attenuated and not significant in men (*P*-interaction between SDB score and sex = 0.01). In a subgroup analysis stratified by BMI categories, the association between summary sleep score and diabetes was consistently present among those with BMI <25 and ≥25 kg/m^2^. The multivariable odds ratio (OR) (95% confidence interval (CI)) of diabetes associated with SDB summary score was 1.36 (1.05–1.77) for BMI < 25 kg/m^2^ and 1.21 (1.09–1.34) for BMI ≥ 25 kg/m^2^ (*P*-interaction between SDB score and BMI categories = 0.2) (data not shown).

 In a supplementary analysis, we examined the association between diagnosed sleep apnea and diabetes. 4.3% of the participants reported having diagnosed sleep apnea. Similar to the individual sleep variables, a significant positive association was observed between diagnosed sleep apnea and diabetes. Compared to those without diagnosed sleep apnea, the multivariable odds ratio (OR) (95% confidence interval (CI)) of diabetes among those with diagnosed sleep apnea was 1.85 (1.19–2.89).

## 4. Discussion

In a representative sample of US adults, we found that markers of SDB including short sleep duration (≤5 h), occasional or frequent snoring (at least 3-4 nights/week), frequent snorting (at least 5 times/week), and frequent daytime sleepiness (at least 5 times/month) were associated with diabetes mellitus independent of age, sex, race-ethnicity, education, smoking, current alcohol consumption, physical activity, BMI, systolic blood pressure, depression, CRP and total cholesterol. Further, an *ad hoc* summary SDB score that provided a measure of the severity of SDB by counting the cooccurrence of these four sleep disturbances showed that, compared to those without any SDB markers, those with three or more SDB markers had more than twofold odds of having diabetes. In subgroup analyses, there were no differences in this association by race-ethnicity or by BMI categories. However, the association between the summary SDB score and diabetes was significant only in women; the association was substantially attenuated and lost statistical significance in men after multivariable adjustment for lifestyle and metabolic risk factors.

 In the current study, we found that SDB, defined by short sleep duration, occasional or frequent snoring, frequent snorting, and frequent daytime sleepiness was associated with diabetes consistent with previous studies [4, 5, 7, 8, 10, 11, 15]. In the Sleep in America poll, daytime sleepiness but not snoring, snorting, and short sleep duration were associated with diabetes among 1506 men and women [8]. Both short and long durations of sleep were shown to be associated with diabetes among men in the Massachusetts Male Aging Study [6], among participants in the first National Health and Nutrition Examination Survey Follow-up Study [10], among a large cohort of participants in the National Health Interview Survey 2004-2005 [7], and men and women in the Sleep Heart Health Study [5]. In contrast, in the Finnish Diabetes Prevention Study, long sleep duration was associated with diabetes among overweight individuals with impaired glucose tolerance [14]. Self-reported snoring was associated with 10-year incidence of diabetes in a large cohort of nurses in the USA [4]. Several studies conducted in the Europe have documented similar associations between self-reported short sleep duration [38], snoring [11, 15], and daytime sleepiness [15] and diabetes. Sleep duration ≤5 h was associated with 12-year incidence of diabetes in Swedish men but not in women [38]. Snoring was associated with 10-year incidence of self-reported diabetes in 2668 men aged 30–69 years in Sweden. Further, joint exposure to both snoring and obesity had a fivefold odds of diabetes compared with their absence [11]. In a cross-sectional study in Finland, self-reported snoring but not daytime sleepiness was associated with diabetes among 593 men and women [15].

 Our study contributes to the existing literature on SDB by demonstrating, in detailed subgroup analyses, that the SDB-diabetes association (1) was consistently present in both non-Hispanic whites and other race-ethnicities, but that (2) there were sex-related differences in this association. First, to our knowledge, this is the first population-based study that compared the association between SDB and diabetes side by side by race-ethnicity as previous studies were conducted either in predominantly white samples or samples involving only African Americans and Hispanic Americans.

 Second, in the subgroup analysis by sex, we found that even though there was a positive association between SDB markers and diabetes in both sexes in the initial age-adjusted model, upon multivariable adjustment for lifestyle and metabolic risk factors, the association was no longer present in men whereas strongly present in women. This finding indirectly suggests that the role of SDB in men is explained by lifestyle and metabolic risk factors such as BMI, lack of physical activity, depression, high CRP, and others where as in women SDB has a residual association that is independent of these factors. Finally, our finding of an independent association in women but not in men is consistent with some [9, 17, 39] but not all previous studies [13]. For example, in the Cardiovascular Health Study, involving 5201 men and women aged ≥65 years in the USA, snoring was associated with diabetes in older women but not in older men [9]. In the MONICA study involving 7905 men and women, snoring was associated with diabetes only in women [17]. In a clinic-based study involving 318 men and women aged 30–69 years in Sweden, SDB defined by polysomnographic measured overnight oxygen desaturation was associated with diabetes in women but not in men [39]. In contrast, in a cross-sectional study involving 1396 men and 1500 women aged 45–74 years in Finland, self-reported SDB was associated with diabetes in middle-aged men but not in women [13].

 Several mechanisms have been proposed to explain the association between SDB and diabetes. Epidemiological studies have shown that short sleep duration is associated with altered glucose metabolism [40], poor glycemic control [41], insulin resistance [40], and markers of inflammation [42]. Snoring with resultant intermittent hypoxia leads to insulin resistance and diabetes [43] through a variety of mechanisms, including activation of the sympathetic nervous system resulting in elevated catecholamine levels [44], activation of the hypothalamic-pituitary-adrenal axis resulting in elevated cortisol levels [45], formation of reactive oxygen species and increased oxidative stress [46], and activation of proinflammatory cytokines [47].

 The large multiethnic sample with rich information on potential confounders and the rigorous methodology of data collection in NHANES are the main strengths of the study. Our study has some limitations. First, assessment of SDB from self-reported sleep measures might have resulted in nondifferential misclassification biasing the association towards null. Second, even though we adjusted for obesity in the multivariable analysis and also performed stratified analysis by BMI categories, it is possible that there is residual confounding by obesity status which may be biasing our results. Third, it is possible that some of the markers that we use to define SDB are biased. For example, there may be gender and/or racial differences in the self-report of snoring. Without polysomnographic examination, it is difficult to avoid measurement error due to self-report. Fourth, our findings are generalizable only to the US noninstitutionalized civilian population; the results may not be representative of institutionalized people or populations outside the USA. Fifth, our cross-sectional study design limits making conclusion on the temporal associations between SDB and diabetes.

 In conclusion, in a nationally representative sample of US adults, we found that sleep duration ≤5 h, occasional or frequent snoring, frequent snorting, and frequent daytime sleepiness were associated with diabetes mellitus independent of potential confounders. This association was significant only in women but consistently present in all ethnic groups.

## Figures and Tables

**Table 1:** Baseline characteristics of the study population*.

Characteristics	Diabetes absent (*n* = 5685)	Diabetes present (*n* = 837)	*P*-value
Women (%)	2782 (50.6)	398 (50.1)	0.454
Age (years)	43.7 ± 0.4	55.4 ± 0.6	<0.0001
Race/Ethnicity (%)			0.0002
Non-Hispanic Whites	2898 (73.5)	321 (63.0)	
Non-Hispanic Blacks	1085 (9.4)	238 (16.8)	
Mexican Americans	1021 (7.8)	185 (9.7)	
Others	681 (9.3)	93 (10.4)	
Education categories (%)			<0.0001
Below high school	1409 (16.1)	335 (24.8)	
High school	1391 (24.3)	197 (26.5)	
Above high school	2885 (59.5)	305 (48.7)	
Smoking (%)			0.184
Never smoker	3045 (52.9)	428 (52.1)	
Former smoker	1284 (22.9)	263 (31.0)	
Current smoker	1356 (24.2)	146 (16.9)	
Current drinker (%)	4056 (76.3)	406 (54.0)	<0.0001
Moderate physical activity (%)	2741 (54.9)	286 (39.0)	<0.0001
Body mass index (%)			<0.0001
Normal weight	1869 (35.2)	114 (12.1)	
Overweight	2036 (34.6)	242 (26.3)	
Obese	1780 (30.1)	481 (61.6)	
Depression (%)	207 (3.1)	52 (6.4)	0.002
Systolic blood pressure (mm Hg)	119.7 ± 0.3	129.3 ± 0.8	<0.0001
C-reactive protein (mg/dL)	0.4 ± 0.01	0.6 ± 0.04	<0.0001
Total cholesterol (mg/dL)	200.7 ± 0.8	194.2 ± 2.1	0.03

*Data presented are number (percentages) or mean values ± standard error (SE), as appropriate for the variable.

**Table 2:** Association between sleep variables and diabetes mellitus.

Sleep variables	No. at risk (*n* = 6522)	Diabetes cases (*n* = 837)	Age-, sex-adjusted odds ratio (95% CI)	Multivariable-adjusted odds ratio* (95% CI)
Sleep duration (hours)				
≤5 hrs	1008	168	1.91 (1.42–2.58)	1.38 (1.01–1.90)
6 hrs	1509	177	1.16 (0.89–1.53)	1.12 (0.80–1.56)
7 hrs	1877	201	1 (referent)	1 (referent)
8 hrs	1719	234	1.09 (0.83–1.43)	1.12 (0.83–1.51)
≥9 hrs	409	57	0.91 (0.55–1.51)	0.79 (0.47–1.34)
*P*-trend			0.0001	0.04
Frequency of snoring				
Never or rarely	3158	298	1 (referent)	1 (referent)
Occasionally	1256	184	1.93 (1.44–2.59)	1.67 (1.23–2.27)
Frequently	2108	355	1.94 (1.58–2.39)	1.44 (1.16–1.79)
*P*-trend			<0.0001	0.0008
Frequency of snorting				
Never or rarely	5718	684	1 (referent)	1 (referent)
Occasionally	421	80	1.23 (0.87–1.73)	0.94 (0.65–1.36)
Frequently	383	73	1.98 (1.48–2.66)	1.37 (1.04–1.79)
*P*-trend			<0.0001	0.09
Frequency of daytime sleepiness				
Never or rarely	3768	481	1 (referent)	1 (referent)
Sometimes	1680	198	1.02 (0.86–1.22)	1.01 (0.81–1.25)
Often and almost always	1074	158	1.51 (1.25–1.83)	1.38 (1.08–1.76)
*P*-trend			0.0002	0.03
SDB summary score				
0	2353	207	1 (referent)	1 (referent)
1	2573	345	1.64 (1.25–2.16)	1.37 (1.04–1.80)
2	1177	203	2.03 (1.60–2.57)	1.45 (1.14–1.85)
≥3	419	82	3.50 (2.55–4.81)	2.04 (1.46–2.87)
*P*-trend			<0.0001	<0.0001

*Adjusted for age (years), sex (men, women), race-ethnicity (non-Hispanic whites, non-Hispanic blacks, Mexican Americans, others), education (< high school, high school, > high school), smoking (never, former, current), current alcohol consumption (absent, present), moderate physical activity (times/week), body mass index (kg/m^2^), depression (absent, present), systolic blood pressure (mm Hg), C-reactive protein (mg/dL) and total cholesterol (mg/dL).

**Table 3:** Association between SDB and diabetes mellitus, by race/ethnicity.

SDB summary score	No. at risk	Diabetes cases	Age-, sex-adjusted odds ratio (95% CI)	Multivariable-adjusted odds ratio* (95% CI)
Non-Hispanic Whites				
0	1209	84	1 (referent)	1 (referent)
1	1242	131	1.73 (1.17–2.55)	1.42 (0.94–2.14)
2	566	74	2.12 (1.48–3.03)	1.48 (1.06–2.07)
≥3	202	32	3.94 (2.40–6.48)	2.21 (1.42–3.45)
*P*-trend			<0.0001	<0.0001
Other race-ethnicities				
0	1144	123	1 (referent)	1 (referent)
1	1331	214	1.40 (1.07–1.83)	1.25 (0.91–1.70)
2	611	129	1.75 (1.22–2.51)	1.38 (0.91–2.10)
≥3	217	50	2.46 (1.52–3.99)	1.73 (0.98–3.06)
*P*-trend			<0.0001	0.03

*Adjusted for age (years), sex (men, women), education (< high school, high school, > high school), smoking (never, former, current), current alcohol consumption (absent, present), moderate physical activity (times/week), body mass index (kg/m^2^), depression (absent, present), systolic blood pressure (mm Hg), C-reactive protein (mg/dL), and total cholesterol (mg/dL).

*P*-interaction (SDB summary score × race-ethnicity) = 0.7.

**Table 4:** Association between SDB and diabetes mellitus, by gender.

SDB summary score	No. at risk	Diabetes cases	Age-adjusted odds ratio (95% CI)	Multivariable-adjusted odds ratio* (95% CI)
Men				
0	1065	109	1 (referent)	1 (referent)
1	1360	180	1.56 (1.17–2.09)	1.38 (1.03–1.86)
2	683	114	1.91 (1.32–2.76)	1.42 (0.96–2.09)
≥3	234	36	1.82 (1.12–2.94)	1.10 (0.59–2.04)
*P*-trend			0.0001	0.25
Women				
0	1288	98	1 (referent)	1 (referent)
1	1213	165	1.68 (1.17–2.43)	1.31 (0.87–2.00)
2	494	89	2.06 (1.51–2.82)	1.48 (0.99–2.20)
≥3	185	46	6.03 (3.65–9.96)	3.68 (2.01–6.72)
*P*-trend			<0.0001	<0.0001

* Adjusted for age (years), race-ethnicity (non-Hispanic whites, non-Hispanic blacks, Mexican Americans, others), education (< high school, high school, > high school), smoking (never, former, current), current alcohol consumption (absent, present), moderate physical activity (times/week), body mass index (kg/m^2^), depression (absent, present), systolic blood pressure (mm Hg), C-reactive protein (mg/dL), and total cholesterol (mg/dL).

*P*-interaction (SDB summary score f × female) = 0.01.
